# Renal arteriovenous fistula after renal biopsy: a case report and literature review

**DOI:** 10.1590/1677-5449.011218

**Published:** 2019-03-25

**Authors:** Sergio Quilici Belczak, Guilherme Delicato Pedroso, Luis Felipe Atihe, Ana Beatriz Furlan Vilela, Raquel Silas Melice, Cicero Benedito, Gustavo Garcia Marques

**Affiliations:** 1 Centro Universitário São Camilo, São Paulo, SP, Brasil.

**Keywords:** arteriovenous fistula, therapeutic embolization, needle biopsy, coil embolization, post-biopsy hematuria, renal Doppler

## Abstract

Acquired renal arteriovenous fistulas (AVF) are rare conditions in which an anomalous connection arises between the arterial and venous systems. Renal AVFs can be classified into three main groups: idiopathic, congenital, and acquired, the last of which are the most common. Incidence has been increasing, due to the growing number of renal biopsies. Although the renal biopsy procedure is relatively safe nowadays, one possible complication is formation of an AVF in the renal vascular territory. Treatment of renal AVF is widely discussed in the literature and a variety of treatment methods can be employed. We report a case of arteriovenous fistula after renal biopsy that was successfully treated with endovascular coil embolization.

## INTRODUCTION

 An arteriovenous fistula (AVF) is an anomalous connection between the arterial and venous systems. 1 The first report of an intrarenal AVF was published by Varela in 1928. 2 The overall prevalence of all types of renal AVF is less than 0.04%, according to estimates by Cho and Stanley. 3

 Three types of renal AVF are described: congenital, accounting for 14 to 27% of these abnormalities 4 ; idiopathic, accounting for 4.8% 5 ; and acquired, accounting for 70 to 80%. 4 ^-^ 6 Acquired and idiopathic renal AVF typically involve large arteriovenous communications. The lower venous vascular resistance of the communication is responsible for reduced blood flow through the renal parenchyma, with renal ischemia and consequent activation of the renin-angiotensin system, causing hypertension and kidney failure. Additionally, the ‘vascular steal’ phenomenon caused by the AVF increases venous return and predisposes patients to high-output heart failure. 7 ^-^ 9

 The elevated incidence of the acquired form of renal AVF has been attributed to the growing number of renal biopsies. However, the condition can also be caused by trauma, inflammation, surgery, tumors, or atherosclerosis. 1 ^,^ 7 ^,^ 10 ^-^ 13 In the majority of cases, the patient does not exhibit symptoms, but there may be hematuria and even hemodynamic changes or loss of renal function in more extreme cases. 1

 Generally, AVFs do not require any intervention whatsoever, because spontaneous closure occurs within 3 months of the biopsy in 95.4% of cases. 1 In cases of major hematuria causing hemodynamic instability, surgical repair is needed. In the past, open surgery was the standard treatment for this condition. However, with the advent of endovascular treatments, high success rates and lower morbidity and mortality can be achieved. Endovascular embolization is now the first line of treatment for AVF cases. Surgery and embolization are successful in 85% of cases. 14 ^,^ 15

## CASE DESCRIPTION

 A 28-year-old patient, with no comorbidities, was admitted to hospital with acute renal failure. A hemolytic-uremic syndrome was suspected and a left renal biopsy was performed via a translumbar puncture. After the procedure, the patient exhibited intense hematuria for 48 hours and became hemodynamically instable, with hemoglobin 5.8 g/% (12.6 g/% at admission) and she required blood transfusion. 

**Figure 1:**
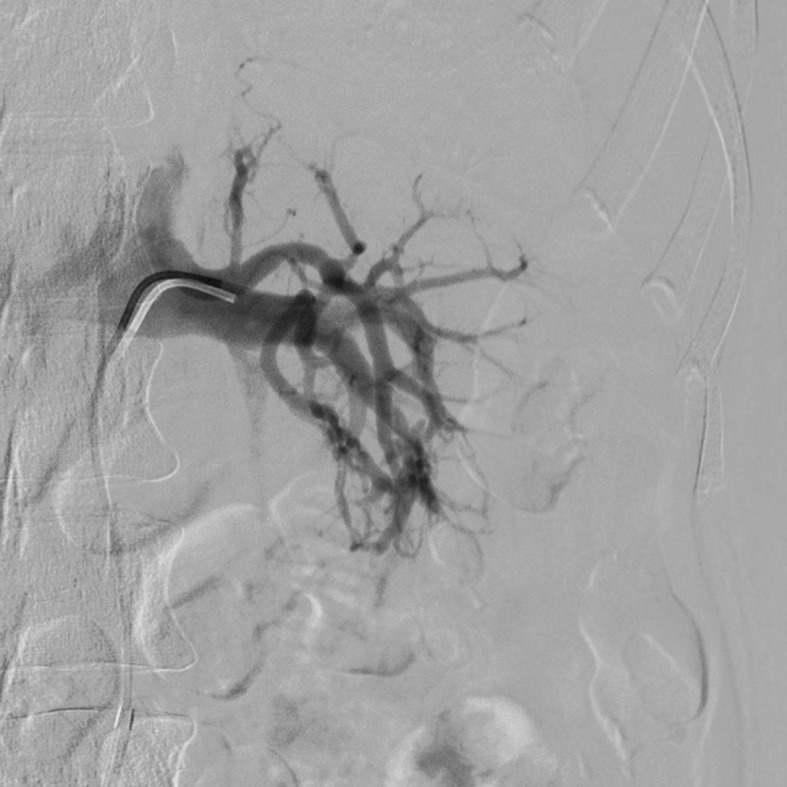
Renal arteriography showing the rapid filling of the renal venous system characteristic of arteriovenous fistulas.

**Figure 2:**
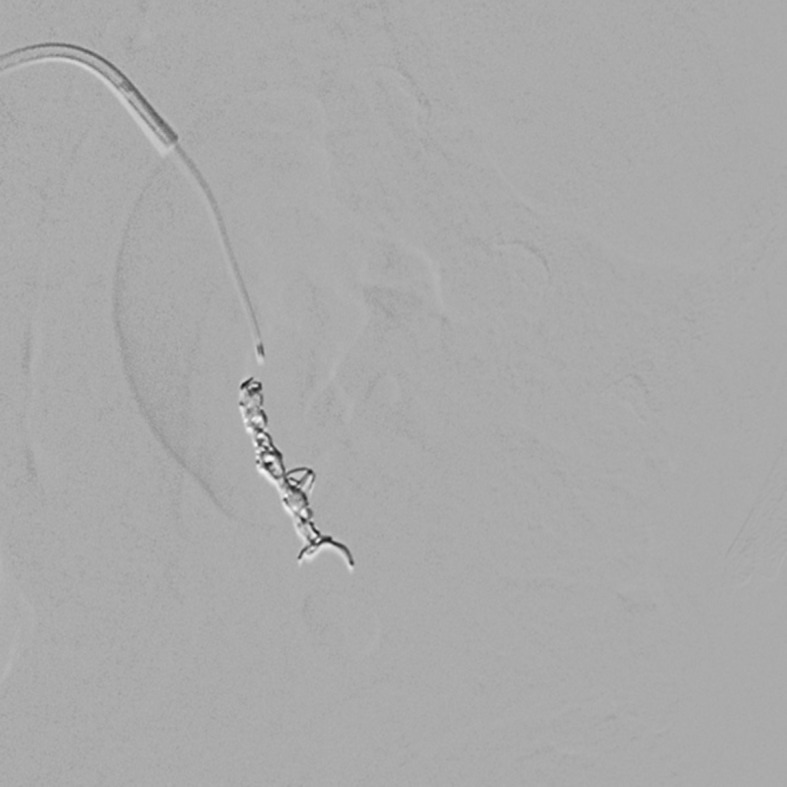
Coil embolization (three units of 3 × 10 mm, three-dimensional, controlled-release coils: Trufill DCS Orbit Complex).

**Figure 3:**
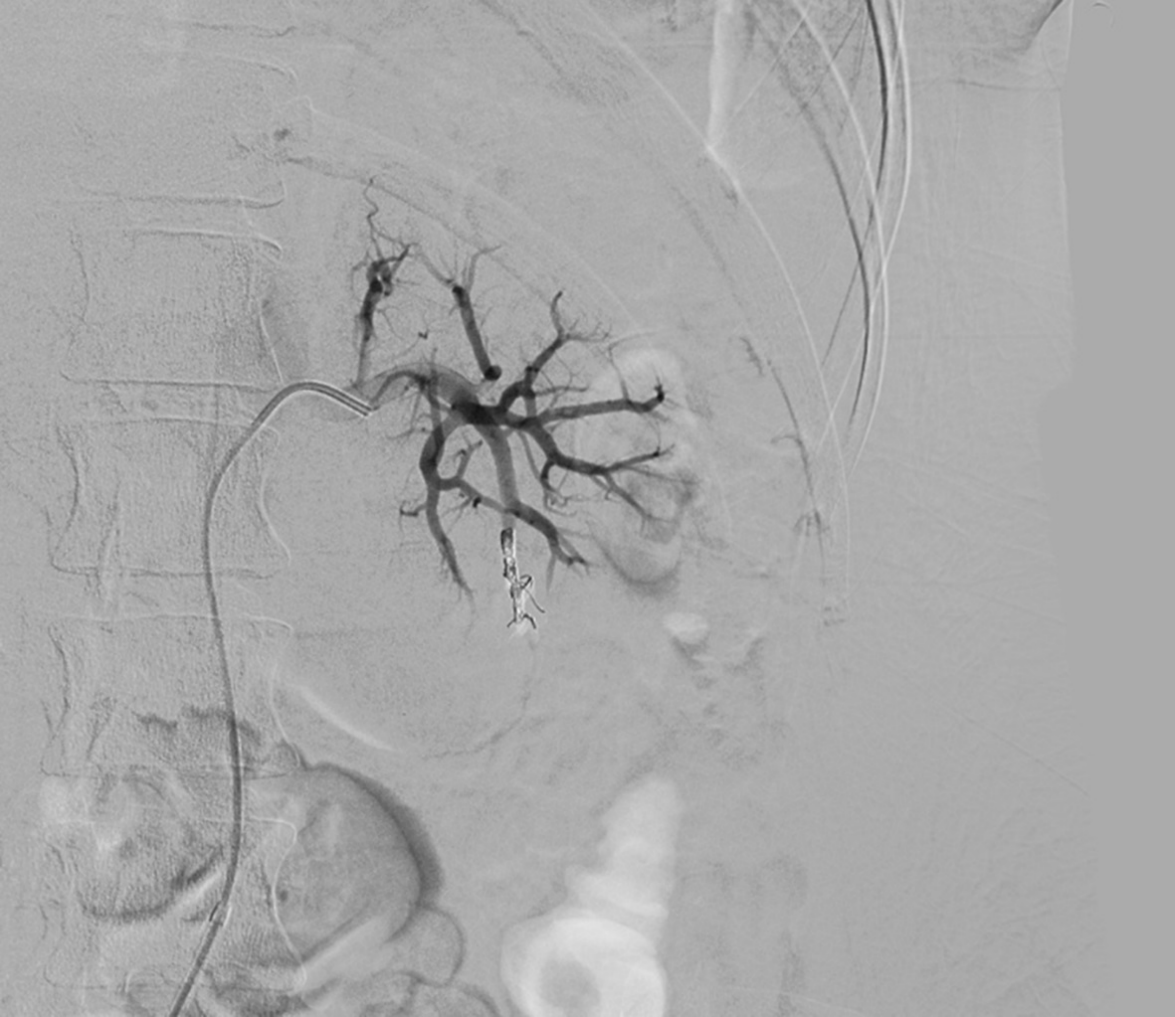
Control angiography showing treatment of the arteriovenous fistula.

 Ultrasound examination of the abdomen and urinary tract was conducted, revealing a large vesical clot. The patient then underwent renal arteriography, which showed rapid filling of the renal venous system, characteristic of a renal AVF ( Figure 1 ). Coil embolization was performed (three units of 3 × 10 mm, controlled-release, three-dimensional coils: Trufill DCS Orbit Complex) ( Figure 2 ). Control angiography showed that the fistula had closed, with normal pelvic renal contrast behavior, and slow venous flow ( Figure 3 ). The hematuria resolved soon after the procedure, and the patient recovered with no further intercurrent conditions and no additional blood transfusions. She remained in the intensive care unit for observation only and was discharged from the critical care department 24 hours later. Her renal function remained unchanged throughout her hospital stay. 

## DISCUSSION

 Renal biopsy is a very useful tool for diagnosis, to determine prognosis, and to guide treatment. Although it is considered safe, since it is an invasive procedure, it is not free from complications, one of which is AVF. These are rare, with incidence rates of 3 to 5% in native kidneys and 10 to 16% in transplanted kidneys, but AVFs can be avoided or their incidence reduced to less than 0.1% if biopsy is conducted with real-time ultrasound guidance and automatic needles. 1 ^,^ 16 ^,^ 17 The procedure is considered a success if, in addition to acquiring sufficient material for diagnostic biopsy, it causes less adverse outcomes for the patient. 18

 An arteriovenous fistula is an anomalous communications between the arterial and venous systems. There are three types of renal AVF: congenital, idiopathic, and acquired. Acquired AVFs are caused by trauma, inflammation, surgery, tumors, atherosclerosis, or percutaneous biopsy and account for 70 to 80% of arteriovenous abnormalities. 1 ^,^ 12 ^,^ 17 Of these, the most common are AVFs associated with percutaneous renal biopsy, those caused by traumas, and those secondary to percutaneous renal surgery. 13 An idiopathic AVF is one that is acquired at some point during life, but has no definite etiologic factor. 4

 Clinical diagnosis of an AVF can be difficult. Signs and symptoms include microscopic and macroscopic hematuria, arterial hypertension refractory to medical treatment, flank pain, and audible sounds in the renal arteries caused by turbulent blood flow. 19 ^,^ 20 The objective of treatment of fistulae and renal arteriovenous malformations is to eradicate the symptoms and hemodynamic effects (arterial hypertension and heart failure), with maximum preservation of functioning renal parenchyma. 12 ^,^ 17 ^,^ 21

 Less aggressive treatment options include blood transfusions. Invasive options that may be necessary in refractory cases and those with major hematuria or hemodynamic instability 6 include cystoscopy, angiography with subsequent embolization with gel-foam or coils (with a success rate of around 85% in patients with acquired fistulae), and surgical nephrectomy. 14

 Described initially in 1973, to deal with AVF associated with biopsy, 22 treatment with percutaneous angiography and embolization is the most effective method and is considered the first-line treatment for these fistulas, achieving success in 70 to 100% of cases. 15 ^,^ 19 ^,^ 23 It is a widely adopted treatment option that can be used as definitive treatment or in an attempt to reduce fistula throughput, and is a less invasive surgical procedure. 12 ^,^ 17 ^,^ 21 The many different embolization agents employed include steel coils, as used in the case described here, balloons, autologous blood clots, absorbable gelatin foam, cyanoacrylate, plastic polymers, and absolute alcohol. 24

 Controlled-release coils offer certain advantages in relation to other methods. Embolization only takes place in the target vessel and, because they offer controlled release, they can be placed exactly where intended. Once they are correctly positioned, they are released. They are also associated with minimal renal ischemia, since they do not close the distal microcirculation and it is possible to precisely occlude only the point at which the artery communicates with the vein. Therefore, this type of material was used in the procedure described here because it offers controlled release without distal occlusion of the vessel, which avoids provoking renal ischemia, occluding the fistula with greater precision. 

 Embolization can be performed via an intra-arterial access or using a combined approach via arterial and venous routes simultaneously. 23 Although small, there is a risk of complications, such as closure of nearby vessels or intact proximal vessels, resulting in notable loss of renal parenchyma, pulmonary embolism, and others. 15 ^,^ 25

 It can therefore be concluded that endovascular intervention for coil embolization is indicated for treatment of the majority of renal AVFs because it is a less invasive method that achieves a high rate of success. 
