# Supplementary material for: Renal arteriovenous fistula after renal biopsy: a case report and literature review
Source: J Vasc Bras. 2019 Mar 25;18:e20180112. doi: 10.1590/1677-5449.011218 (PMC6582767; doi:10.1590/1677-5449.011218)
Supplement: Translation

# Translation: Fístula arteriovenosa renal pós-biópsia renal: relato de caso e revisão de literatura‏

**Authors:** Sergio Quilici Belczak, Guilherme Delicato Pedroso, Luis Felipe Atihe, Ana Beatriz Furlan Vilela, Raquel Silas Melice, Cicero Benedito, Gustavo Garcia Marques

**Affiliations:** 1 Centro Universitário São Camilo, São Paulo, SP, Brasil.

**Keywords:** fístula arteriovenosa, embolização terapêutica, biópsia por agulha, embolização com molas, hematúria pós-biópsia, Doppler renal

## Abstract

Fístulas arteriovenosas (FAVs) renais adquiridas são raras, correspondendo a uma conexão anômala entre o sistema arterial e o sistema venoso. As FAVs renais se dividem em três grandes grupos: idiopáticas, congênitas e adquiridas, sendo as últimas as mais comuns. Atualmente, têm incidência aumentada em decorrência do crescente número de biópsias renais. Apesar de, atualmente, o procedimento de biópsia renal ser relativamente seguro, ele carrega como complicação a formação de FAV no território vascular renal. O tratamento de FAV renal é amplamente discutido na literatura e diversas modalidades terapêuticas podem ser aplicadas. Apresentamos um caso de FAV pós-biópsia renal que foi submetida a tratamento endovascular com sucesso mediante embolização com molas.

## INTRODUÇÃO

 A fístula arteriovenosa (FAV) corresponde a uma conexão anômala entre o sistema arterial e o venoso 1 . A primeira FAV intrarrenal foi relatada por Varela em 1928 2 . A prevalência de qualquer tipo de FAV renal é menor que 0,04%, de acordo com as estimativas de Cho e Stanley 3 . 

 São descritas três variedades de FAV renal: congênita, correspondendo a 14 a 27% das anormalidades 4 ; idiopática, correspondendo a 4,8% 5 ; e adquirida, correspondendo a 70 a 80% 4 ^-^ 6 . A FAV renal adquirida e a idiopática são caracterizadas por grandes comunicações arteriovenosas. A reduzida resistência vascular venosa dessas comunicações causa diminuição do fluxo sanguíneo através do parênquima renal, acarretando isquemia renal e consequente ativação do sistema renina-angiotensina, que propicia o surgimento de hipertensão e insuficiência renal. Além disso, esse fenômeno de “roubo vascular”, que ocorre devido à FAV, aumenta o retorno venoso e predispõe à insuficiência cardíaca de alto débito 7 ^-^ 9 . 

 A forma adquirida da FAV renal tem sido atribuída, devido a sua alta incidência, ao número crescente de biópsias renais. Porém, também pode ser decorrente de trauma, inflamação, cirurgia, tumor ou aterosclerose 1 ^,^ 7 ^,^ 10 ^-^ 13 . Essa condição, na maioria dos casos, não ocasiona sintomas no paciente, mas pode gerar hematúria e até levar a alterações hemodinâmicas ou perda da função renal em casos mais extremos 1 . 

 Geralmente, as FAVs não necessitam de qualquer intervenção, pois em 95,4% dos casos há o fechamento espontâneo dentro de três meses após a biópsia 1 . Em casos de hematúria franca ocasionando instabilidade hemodinâmica, há a necessidade de correção cirúrgica. Anteriormente, a cirurgia aberta era a terapia-padrão para essa condição. Entretanto, com o advento da terapia endovascular, foi documentada uma alta taxa de sucesso aliada a uma menor taxa de morbimortalidade. A embolização endovascular representa atualmente a primeira linha de tratamento para os casos de FAV. A cirurgia e a embolização obtêm sucesso em 85% dos casos 14 ^,^ 15 . 

## DESCRIÇÃO DO CASO

 Paciente de 28 anos, sem comorbidades, foi internada por quadro de insuficiência renal aguda. Suspeitou-se de síndrome hemolítico-urêmica e realizou-se biópsia renal esquerda por punção translombar. Após o procedimento, a paciente apresentou quadro de hematúria intensa por 48 horas, evoluindo com instabilidade hemodinâmica, hemoglobina de 5,8 g/% (valor de entrada: 12,6 g/%) e necessidade de transfusão sanguínea. 

 Realizou-se ultrassonografia de abdome e vias urinárias, que evidenciou grande coágulo vesical. A paciente foi submetida, então, a arteriografia renal, que revelou rápido enchimento do sistema venoso renal, caracterizando FAV renal ( Figura 1 ). Realizou-se embolização com molas (três unidades de molas tridimensionais de liberação controlada, 3 × 10 mm, Trufill DCS Orbit Complex) ( Figura 2 ). A angiografia de controle mostrou fechamento da fístula com contrastação normal da pelve renal e lento escoamento venoso ( Figura 3 ). Já a hematúria cessou logo após o procedimento, e a paciente evoluiu sem novas intercorrências nem necessidade de transfusão sanguínea. Permaneceu na unidade de terapia intensiva apenas para observação, sendo liberada desse setor após 24 horas. Sua função renal não se alterou em nenhum momento durante a internação. 

**Figura 1:** Arteriografia renal com rápido enchimento do sistema venoso renal, caracterizando fístula arteriovenosa.

**Figura 2:** Embolização com molas (três unidades de molas tridimensionais de liberação controlada, 3 × 10 mm, Trufill DCS Orbit Complex).

**Figura 3:** Angiografia de controle evidenciando tratamento da fístula arteriovenosa.

## DISCUSSÃO

 A biópsia renal representa uma ferramenta muito útil para realizar diagnósticos, determinar prognósticos e orientar tratamentos. Apesar de ser considerada segura, por se tratar de um procedimento invasivo, não está isenta de complicações, sendo uma delas as FAVs. Embora sejam raras, com uma incidência de 3 a 5% em rins nativos e de 10 a 16% em rins transplantados, as FAVs podem ser evitadas e/ou ter sua incidência diminuída para menos de 0,1% ao ser realizada de forma guiada por ultrassonografia em tempo real e com agulhas automatizadas 1 ^,^ 16 ^,^ 17 . Considera-se um procedimento de sucesso aquele que, além de obter material suficiente para biópsia diagnóstica, implique em menos desfechos adversos ao paciente 18 . 

 As FAVs são comunicações anômalas entre o sistema arterial e o venoso. Existem três tipos de FAV renal: congênita, idiopática e adquirida. A FAV adquirida é decorrente de trauma, inflamação, cirurgia, tumor, aterosclerose ou biópsia percutânea, representando de 70 a 80% das anormalidades arteriovenosas 1 ^,^ 12 ^,^ 17 . Entre essas, as mais comuns são as FAVs associadas à biópsia renal percutânea, as produzidas por traumatismo e as secundárias à cirurgia renal percutânea 13 . A FAV idiopática é adquirida ao longo da vida, mas não possui um fator etiológico definido 4 . 

 O diagnóstico clínico de FAV pode ser difícil. Os sinais e sintomas incluem hematúria microscópica e macroscópica, hipertensão arterial refratária ao tratamento médico, dor no flanco e ruídos nas artérias renais devido a um fluxo sanguíneo turbulento 19 ^,^ 20 . O tratamento das fístulas e malformações arteriovenosas renais tem por objetivo erradicar os sintomas e os efeitos hemodinâmicos (hipertensão arterial e insuficiência cardíaca), com máxima preservação do parênquima renal funcionante 12 ^,^ 17 ^,^ 21 . 

 Como terapia menos agressiva, conta-se com a hemotransfusão. Já as opções invasivas, que podem ser necessárias em caso de refratariedade, hematúria de grande monta ou instabilidade hemodinâmica 6 , são a cistoscopia, a angiografia com subsequente embolização com gel-*foam* ou *coil*, cujo sucesso é de cerca de 85% dos pacientes com fístulas adquiridas, ou a nefrectomia cirúrgica 14 . 

 Descrito inicialmente em 1973 para associação entre FAV e biópsia 22 , o tratamento com angiografia percutânea e embolização é o mais efetivo, considerado a terapia de primeira linha para essas fístulas, com sucesso em 70 a 100% dos casos 15 ^,^ 19 ^,^ 23 . É uma opção de tratamento bastante difundida, podendo ser utilizada como tratamento definitivo ou na tentativa de redução da fístula, caracterizando um procedimento cirúrgico menos invasivo 12 ^,^ 17 ^,^ 21 . Dos diversos agentes utilizados estão as molas de aço, usadas no caso relatado, balões, coágulos sanguíneos autólogos, espuma de gelatina absorvível, cianoacrilato, polímeros plásticos e álcool absoluto 24 . 

 A utilização de mola de liberação controlada tem algumas vantagens em relação a outros métodos. A embolização ocorre somente no vaso desejado e, pelo fato de ser um destacamento controlado, vai exatamente para o local almejado. Estando devidamente alocada, é então liberada. Além disso, apresenta mínima isquemia renal, uma vez que não fecha a microcirculação distal, e consegue ocluir com precisão somente o sítio de comunicação da artéria com a veia. Assim, neste procedimento foi utilizado esse tipo de material devido a sua liberação controlada sem oclusão distal do ramo para não promover isquemia renal, ocluindo com maior precisão o trajeto fistuloso. 

 A embolização pode ser por acesso intra-arterial ou combinado, utilizando a via arterial e as vias venosas simultaneamente 23 . Apesar de pequeno, existe o risco de complicações, como fechamento de vasos proximais ou dos vasos intactos próximos, resultando em perda notável do parênquima renal, embolia pulmonar, entre outras 15 ^,^ 25 . 

 Portanto, conclui-se que a intervenção endovascular de embolização com molas é bem indicada para o tratamento da maioria das FAVs renais, pois se trata de um método menos invasivo que apresenta elevados índices de sucesso.
